# Maternal BMI, IGF-I Levels, and Birth Weight in African American and White Infants

**DOI:** 10.1155/2013/191472

**Published:** 2013-06-03

**Authors:** Adriana C. Vidal, Amy P. Murtha, Susan K. Murphy, Kimberly Fortner, Francine Overcash, Nikki Henry, Joellen M. Schildkraut, Michele R. Forman, Wendy Demark-Wahnefried, Joanne Kurtzberg, Randy Jirtle, Cathrine Hoyo

**Affiliations:** ^1^Department of Obstetrics and Gynecology and Program of Cancer Detection, Prevention and Control, Duke University School of Medicine, Durham, NC 27710, USA; ^2^Division of Maternal-Fetal Medicine, Department of Obstetrics and Gynecology, Duke University School of Medicine, Durham, NC 27710, USA; ^3^Division of Gynecologic Oncology, Department of Obstetrics and Gynecology, Duke University School of Medicine, Durham, NC 27710, USA; ^4^Division of Maternal Fetal Medicine, Department Obstetrics and Gynecology, Vanderbilt University School of Medicine, Nashville, TN 37232, USA; ^5^Department of Obstetrics and Gynecology, Duke University School of Medicine, Durham, NC 27710, USA; ^6^Duke University School of Medicine, Durham, NC 27710, USA; ^7^Department of Community and Family Medicine and Program of Cancer Detection, Prevention and Control, Duke University Medical Center, Durham, NC 27710, USA; ^8^Department of Epidemiology, The University of Texas at Austin, Austin, TX 78712, USA; ^9^Department of Nutrition Sciences and UAB Comprehensive Cancer Center, University of Alabama at Birmingham, Birmingham, AL 35294, USA; ^10^Carolina Cord Blood Bank, Duke University Medical Center, Durham, NC 27710, USA; ^11^Department of Radiation Oncology, Duke University Medical Center, Durham, NC 27710, USA; ^12^Division of Epidemiology, Department of Obstetrics and Gynecology, Duke University School of Medicine, Durham, NC 27710, USA

## Abstract

At birth, elevated IGF-I levels have been linked to birth weight extremes; high birth weight and low birth weight are risk factors for adult-onset chronic diseases including obesity, cardiovascular disease, and type 2 diabetes. We examined associations between plasma IGF-I levels and birth weight among infants born to African American and White obese and nonobese women. Prepregnancy weight and height were assessed among 251 pregnant women and anthropometric measurements of full term infants (≥37 weeks of gestation) were taken at birth. Circulating IGF-I was measured by ELISA in umbilical cord blood plasma. Linear regression models were utilized to examine associations between birth weight and high IGF-I, using the bottom two tertiles as referents. Compared with infants with lower IGF-I levels (≤3rd tertile), those with higher IGF-I levels (>3rd tertile) were 130 g heavier at birth, (*β*-coefficient = 230, se = 58.0, *P* = 0.0001), after adjusting for gender, race/ethnicity, gestational age, delivery route, maternal BMI and smoking. Stratified analyses suggested that these associations are more pronounced in infants born to African American women and women with BMI ≥30 kg/m^2^; the cross product term for IGF-I and maternal BMI was statistically significant (*P* ≤ 0.0004). Our findings suggest that the association between IGF-I levels and birth weight depends more on maternal obesity than African American race/ethnicity.

## 1. Introduction

Low birth weight (LBW) and high birth weight (HBW) are both important indicators of suboptimal intrauterine development and have been linked to risk of several chronic diseases later in life. HBW has been associated with childhood and adult obesity [1] and some cancers, including breast [2] and prostate cancer [3], whereas LBW is a consistently identified risk factor for cardiovascular disease (CVD) [4, 5] and type 2 diabetes (T2D) [6, 7]. 

IGF-I is a mitogenic and antiapoptotic paracrine growth factor expressed in all fetal organs [8, 9], and is essential in fetal and neonatal growth, differentiation and development [10–14]. Several lines of evidence suggest that IGF-I levels are associated with birth weight [15–26]; furthermore higher IGF-I levels are associated with higher BW, but not with lower birth weight [16, 17, 26]. In adulthood, elevated concentrations of IGF-I are associated with an increased risk of obesity and many cancers, including breast, lung, head and neck, colorectal, pancreas, synovial sarcoma, and prostate cancer [26–31]. Although concentrations of circulating IGF-I levels vary considerably by race/ethnicity and maternal prepregnancy obesity, few studies included African American women [24–26], an ethnic group that has an almost 9% higher BMI than European-American women [32]. Furthermore, studies examining both maternal obesity, an increasingly common condition, and race/ethnicity are few [33–35]. Herein, we examine associations between IGF-I levels in relation to body size at birth in a cohort of infants born to obese and non-obese African American and White women.

## 2. Subjects and Methods

*Ethics Statement.* The study protocol was approved by the Duke University Institutional Review Board (IRB). We obtained written informed consent from all participants involved in our study. We also obtained informed written consent from carers or guardians on the behalf of the minors/children participants involved in our study.

### 2.1. Study Participants

Pregnant women were enrolled as part of the Newborn Epigenetics STudy (NEST), a prospective study of women and their infants. Detailed methods for enrollment of study participants have been reported elsewhere [36]. Briefly, pregnant women who were 18 years and older and who planned to give birth at Duke University or Durham Regional Hospitals were recruited from Durham County, NC prenatal clinics. HIV-1 infected women were excluded. Of the 1101 women approached, 940 (85%) consented and were enrolled; most of the enrolled sample (81%) was successfully followed to delivery (*n* = 757). IGF-I analyses were performed on the first 251 women and had a gestational age ≥37 weeks. Data from these participants were comparable to participants in whom IGF-I was not measured with respect to maternal age, gestational age, race/ethnicity, and maternal prepregnancy BMI.

### 2.2. Data Collection

Interviewer- or self-administered questionnaires were obtained at the time of enrollment and medical records during pregnancy were abstracted at parturition. Data collected using questionnaires included sociodemographic characteristics, reproductive factors, and lifestyle factors such as cigarette smoking and self-reported prepregnancy anthropometric measurements. Information on anthropometric measurements during pregnancy, birth outcomes, including birth weight and length, was abstracted from medical records. Umbilical cord blood specimens were also collected at birth, as previously described [36, 37]. 

### 2.3. IGF-I Cord Blood Levels

Plasma concentrations of IGF-I were measured using microplate enzyme-linked immunosorbent assays (ELISA, Diagnostic Systems Laboratories, Webster, TX, USA) according to the manufacture instructions, and triplicate assays per sample were performed. Absorbance was read using a microplate reader (DSL, Webster, TX, USA). The plasma IGF-I concentrations ranged from 3.65 ng/L to 418.36 ng/L, and assay sensitivity was 2.2 ng/mL.

### 2.4. Statistical Analysis

Infant birth weight was normally distributed and was used as a continuous variable. Because both high birth weight (HBW) and low birth weight (LBW) have been linked to maternal obesity [1], we also analyzed birth weight as a categorical variable: LBW, <2500 g, normal birth weight, 2500–4000 g, and HBW, >4000 g. We used chi-squared tests to examined distribution of factors that have been previously associated with birth weight >4000 g and <2500 g in relation to maternal age (categorized as 18–25, 26–30, and >30 years); prepregnancy mothers' body mass index (BMI) (categorized as <30 and ≥30 kg/m^2^); race/ethnicity (categorized as White, African American, and other); and maternal education (categorized as less than college, college, and graduate level education), maternal smoking, and infant gender. Although some of these factors were not statistically significantly associated with BW in this study, we adjusted for maternal age, BMI, race, education, delivery route, gestational age at delivery, maternal smoking during pregnancy, and infant gender, as they are associated with BW and IGF-I in the population. The distribution of IGF-I levels departed from normality and thus they were categorized into tertiles. We then dichotomized at the top tertile (67th percentile) and compared this tertile (high IGF-I; >67th percentile) to the remainder (low IGF-I; ≤67th percentile). Linear regression models were used to examine the extent to which IGF-I levels differed by birth weight, as described in [36]. All statistical analyses were conducted in SAS v9.2 (SAS Institute Inc., Cary, NC, USA).

## 3. Results

Approximately 4% of the 251 newborns were born <2500 g and ~8% were born >4000 g. Mean gestational age at delivery was 39 weeks (SD = 1.14).  Table 1 summarizes the characteristics of the study participants in relation to birth weight. Most low birth weight infants (<2500 g) were born to African Americans (89%), cigarette smokers (before and during pregnancy, 80%), and those with a lower educational achievement (67%); however the differences were not statistically significant. LBW were however comparable to infants born 2500–4000 g with respect to maternal age. HBW infants were comparable to those born 2500–4000 g with respect to education and maternal smoking, although they were more frequently born to white women and those with a BMI ≥30 before pregnancy. No statistically significant differences in IGF-I levels were found between birth weight <2500 g infants and birth weight >4000 g infants; however birth weight <2500 g infants were born two weeks earlier than infants born 2500–4000 g and birth weight >4000 g infants (*P* = 0.006) (Table 1). 

 Median IGF-I concentration in newborns was 35.55 ng/L (IQR, 17.35–65.68) with a mean of 58.92 ng/L. Birth weight ranged between 2000 and 5160 g (mean = 3315 g, SD = 486). Infants with higher levels of IGF-I (>3rd tertile) were born slightly heavier than infants with lower IGF-I concentrations (≤3rd tertile), although the differences were not statistically significant. Infants with higher IGF-I levels were comparable to those with lower levels with respect to maternal age (*P* = 0.65), race (*P* = 0.83), maternal education (*P* = 0.38), maternal smoking (*P* = 0.63), obesity (*P* = 0.28), and gestational age (*P* = 0.91) (Table 2), although they may differ by delivery route (*P* = 0.14).

Table 3 shows coefficient estimates (*β*'s) and their standard errors (SEs) for five multivariate linear regression models of birth weight on IGF-I levels, in which IGF-I ≤67th percentile concentrations or ≤3rd tertile served as referents; one for all participants, one restricted to African Americans, one restricted to Whites, one restricted to maternal BMI <30, and one restricted to maternal BMI ≥30. The first model shows that after adjusting for infant gender, race, gestational age at delivery, delivery route, maternal BMI and smoking during pregnancy, infants in the upper tertile of IGF-I were larger at birth (*β* = 230.46, se = 58.0, *P* = 0.0001), corresponding to 130 g increased birth weight in infants born to those with high IGF-I levels (Table 3). We also found that this association was slightly higher in African American infants (*P* = 0.002) compared to White infants (*P* = 0.02). However, the cross-product term for race and IGF-I was not significant (*P* = 0.5). Since the prevalence of BMI ≥30 in African American women in our study is higher (67%) compared to Whites (24%) (*P* < 0.0001), we examined the role of maternal BMI on the relationship between IGF-I levels and birth weight. The pair of models restricted by BMI revealed that the association of IGF-I levels and infant size was limited to women with a BMI ≥30 (*β* = 617.78, se = 134.68, *P* < 0.0001). We found a strong positive association in infants born to obese women; the cross-product term for maternal BMI and IGF-I was *P* = 0.0004. 

## 4. Discussion 

Our key finding was a positive association among term infants with elevated IGF-I levels and an increased risk of higher birth weight. This positive association was most apparent in infants born to obese women. High birth weight and high IGF-I levels—a potent mitogen—have been linked to CVD, T2D, and some cancers [2–7]. Importantly, our data also demonstrate that, after accounting for maternal obesity before pregnancy, the influence of ethnicity/race on the association between IGF-I and birth weight (an important risk factor for common chronic diseases) is dramatically diminished. 

The extent to which the positive association between IGF-I at birth and early postnatal growth that is maintained during life course to adulthood is still unclear, although a few studies report that elevated IGF-I levels measured at birth are associated with age 4-5 year obesity [38, 39].

IGF-I levels found in this study (median = 36 ng/L, ICR 17 ng/L–66 ng/L) are slightly lower than those reported in previous studies [25]; however our findings that higher IGF-I levels at birth are associated with increased birth weight are consistent with those of others [18], and with reports which found positive associations between both placental [40] and cord plasma IGF-I concentrations and lower birth weight infants, compared with IGF-I concentrations in normal birth weight infants [19, 41–44]. Our findings however contrast with those of Wang et al. [45] and Deiber et al. [46], who found low birth weight associated with higher IGF-I in populations of infants born to White women. Divergent findings may be due, in part, to lower incidence of maternal obesity among European women; the strongest association between IGF-I and birth weight was found in infants born to obese women. It is speculated that such differences may involve the IGF axis through IGF-I and growth hormone (GH) signaling, which differ by obesity [21, 23, 47]. 

These race/ethnic similarities exist despite IGF-I concentrations being generally lower in African American than in White neonates born with normal weight, as shown in male [25] and in female newborns [26], and in studies performed in adults [33]. 

 Our findings that elevated IGF-I levels are associated with increased birth weight in infants born to obese mothers, are consistent with at least three other studies comprising European, Maori, South Asian, and Pacific Islands' cohorts [33, 47, 48], supporting the hypothesis that IGF-I and insulin resistance perhaps driven by genetic factors may play a role in fetal growth. IGF-I levels in pregnant women have been positively correlated with maternal body weight [33, 49]. Maternal insulin sensitivity has been proposed as an important endogenous environmental factor in fetal size [50]. Maternal hyperglycemia, a correlate of obesity, even considered in the normal range, may affect fetal weight [51]. Together, these findings suggest that the IGF axis may play a key role in fetal overgrowth and maternal obesity may be an important driver in placental IGF-axis concentrations, and thus increasing the risk for higher birth weight and infant obesity in childhood. 

The main limitation of this study was relatively small sample size for analyses restricted to maternal BMI (BMI < 30 versus BMI ≥ 30); however, findings are consistent with the expected role of IGF-I, and IGF-I values themselves (range, median, and skewness of IGF-I levels) and the magnitude of the positive associations with higher birth weight and growth are similar to previous reports [25, 26]. Also, we relied on a single measure of IGF-I levels at delivery; however it was not feasible to obtain IGF-I levels before birth in these infants. However, at least postnatally, interindividual variation seems stable and most studies relating cord blood IGF-I protein concentrations and birth weight reported single measurements at birth [18, 52], and findings are similar. Consideration should also be given to the fact that infant's race/ethnicity is the self-reported mother's race/ethnicity and the study is predominantly African Americans and Whites. Future studies should use ancestral markers to further clarify race specific associations. Another limitation was that we did not measure other components of the IGF axis, including growth hormone, insulin, IGF binding proteins (IGFBP1-5), and receptors' (IGF-1R and 2R) concentrations in the newborns, to further establish the potential implication of other components of the IGF system in fetal growth. However the assay used measured total cord blood IGF-I in circulation. Despite these limitations, our findings support the hypothesis that elevated IGF-I is an important growth factor in early development that has been linked to a number of common chronic diseases, regardless of race/ethnicity. 

In summary, we found that elevated IGF-I levels in newborns increase the risk of higher birth weight in African American and White infants. Our findings also suggest that maternal BMI ≥30 may modify positive associations between IGF-I and higher birth weight, supporting the idea that maternal obesity may be key in increasing the risk of higher birth weight in the newborns, an important risk factor for several chronic diseases, including cancer. These findings suggest that the potential utility of IGF-I levels as a marker of future obesity in the offspring is limited to those born to obese women. As the prevalence of maternal obesity continues to increase, larger multiethnic studies with followup at shorter intervals that allow for maternal obesity-specific analyses are required to further elucidate the nature of the positive association between IGF-I levels and higher birth weight.

## Figures and Tables

**Table 1:** Characteristics of study participants.

Characteristic	Birth weight			
	<2500 g (*n* = 10)	2500–4000 g (*n* = 218)	>4000 (*n* = 21)	*P* value
Maternal age at enrollment (yrs)				0.79
18–25 (*n* = 78)	4 (40%)	67 (31%)	6 (28.5%)	
26–30 (*n* = 76)	4 (40%)	65 (30%)	6 (28.5%)	
>30 (*n* = 97)	2 (20%)	86 (39%)	9 (43%)	
Maternal BMI before pregnancy				0.17
<30 (*n* = 159)	7 (87.5%)	140 (71%)	11 (55%)	
≥30 (*n* = 66)	1 (12.5%)	56 (29%)	9 (45%)	
Mean BMI	26.61 (SD = 4.8)	28.4 (SD = 9.4)	31.3 (SD = 12.3)	0.37
Race/ethnicity				0.1
African American (*n* = 126)	8 (89%)	110 (51%)	8 (38%)	
White (*n* = 97)	1 (11%)	85 (39%)	9 (43%)	
Other (*n* = 25)	0 (0%)	21 (9.7%)	4 (19%)	
Education				0.18
<High School HS Graduate/GED (*n* = 95)	6 (67%)	79 (39%)	9 (43%)	
Some College/College Graduate/	3 (33%)	136 (63%)	12 (57%)
Graduate education (*n* = 152)			
Maternal smoking				0.14
Never smoked (*n* = 135)	2 (20%)	120 (55%)	13 (62%)	
Smoked during pregnancy (*n* = 42)	3 (30%)	36 (17%)	1 (5%)	
Quit smoking before pregnancy (*n* = 74)	5 (50%)	62 (28%)	7 (33%)	
Gender of infant				0.12
Male (*n* = 105)	5 (50%)	86 (39%)	13 (62%)	
Female (*n* = 146)	5 (50%)	132 (61%)	8 (38%)	
Delivery route				0.75
Vaginal (*n* = 149)	7 (70%)	131 (61%)	10 (48%)	
Caesarean section (*n* = 101)	3 (30%)	86 (39%)	11 (52%)	
Gestational age at delivery in weeks				0.006
Mean (SD)	37 (0.22)	39 (1.11)	39 (0.93)	
IGF-I				0.12
≤3rd Tertile (*n* = 170)	5 (50%)	153 (70%)	11 (52%)	
>3rd Tertile (*n* = 81)	5 (50%)	65 (30%)	10 (48%)	

Numbers do not necessarily add up due to missing values.

**Table 2:** Characteristics of study participants by Infant IGF-I levels (ng/L).

	IGF-I Levels		*P* value
	≤3rd tertile (*n* = 170)	>3rd tertile (*n* = 81)	
Maternal age at enrollment (yrs)			0.65
18–25 (*n* = 78)	50 (29%)	28 (35%)	
26–30 (*n* = 76)	54 (32%)	22 (27%)	
>30 (*n* = 97)	66 (68%)	31 (38%)	
Maternal BMI			0.28
<30 (*n* = 159)	104 (68%)	55 (75%)	
≥30 (*n* = 66)	48 (32%)	18 (25%)	
Race			0.83
African American (*n* = 126)	86 (51%)	40 (500%)	
White (*n* = 9)	64 (38%)	33 (4111%)	
Other (*n* = 25)	18 (11%)	7 (9%)	
Education			0.38
<High School HS Graduate/GED (*n* = 95)	67 (40%)	28 (35%)	
Some College/College Graduate/	99 (60%)	53 (65%)
Graduate Education (*n* = 152)		
Delivery route			0.14
Vaginal (*n* = 149)	94 (55%)	55 (68%)	
Caesarean section (*n* = 101)	75 (44%)	26 (32%)	
Maternal smoking			0.63
Never smoked (*n* = 135)	95 (56%)	40 (49%)	
Smoked during periconception (*n* = 42)	27 (16%)	15 (19%)	
Quit smoking before periconception (*n* = 74)	48 (28%)	26 (32%)	
Gender of infant			0.29
Male (*n* = 105)	75 (44%)	30 (37%)	
Female (*n* = 146)	95 (56%)	51 (63%)	
Mean Gestational age in weeks (SD)*	39 (1.16)	39 (1.08)	0.91
Mean Birth weight (SD)*	3273 (460.6)	3414.74 (523.5)	0.56

Numbers do not necessarily add up due to missing values.

*Unadjusted.

**Table 3:** ^
1^Adjusted regression coefficients and standard errors (SE) for the associations between newborn IGF-I and Birth Weight in *n* = 251 African American and White infants.

			Birth weight		
	All (*n* = 251)	African American (*n* = 126)	Whites (*n* = 97)	Maternal BMI < 30 (*n* = 159)	Maternal BMI ≥ 30 (*n* = 66)
	*β*-coefficient, SE, *P* value	*β*-coefficient, SE, *P* value	*β*-coefficient, SE, *P* value	*β*-coefficient, SE, *P* value	*β*-coefficient, SE, *P* value
High IGF-I**	230.46, 58.0, 0.0001	280.98, 90.24, 0.002	202.05, 89.72, 0.02	122.31, 62.65, 0.05	617.78, 134.68, <0.0001
Female infants***	−51.7, 55.68, 0.35	67.36, 80.54, 0.40	−172.47, 90.08, 0.06	−137.68, 62.1, 0.03	146.20, 111.10, 0.19
White Race****	240.60, 63.07, 0.0002	—	—	202.11, 46.26, <0.0001	35.21, 87.27, 0.68
High maternal BMI (≥30)^*∧*^	201.68, 62.55, 0.001	261.81, 82.95, 0.002	161.64, 125.81, 0.2	—	—
Gestational age at delivery^*∧∧*^	205.92, 23.84, <0.0001	206.58, 35.92, <0.0001	172.63, 37.79, <0.0001	213.25, 24.9, <0.0001	187.13, 54.47, 0.001
Smoking during Pregnancy^*∧∧∧*^	−8.11, 31.02, 0.79	−5.90, 47.40, 0.90	−39.63, 47.26, 0.40	−46.36, 33.28, 0.16	2.36, 68.16, 0.97
Delivery route^*∧∧∧∧*^	151.57, 56.58, 0.008	229.73, 84.34, 0.008	74.06, 86.27, 0.40	146.23, 62.58, 0.03	178.73, 111.78, 0.11

^1^Factors mutually adjusted for each other: IGF-I levels, infant gender, race, maternal BMI, maternal smoking, delivery route, and gestational age at delivery, to predict birth weight.

**Referents are individuals with lower (≤3rd Tertile) IGF-I at delivery.

***Referents are male infants.

****Referents are African Americans.

^*∧*^Referents are BMI < 30.

^*∧∧*^Referents are lower gestational age.

^*∧∧∧*^Referents are nonsmoking mothers.

^*∧∧∧∧*^Referents are vaginal delivery infants.
